# Decades-long phylogeographic issues: complex historical processes and ecological factors on genetic structure of alpine plants in the Japanese Archipelago

**DOI:** 10.1007/s10265-022-01377-w

**Published:** 2022-02-15

**Authors:** Hajime Ikeda

**Affiliations:** Institute of Plant Science and Resources, Okayama University, 2-20-1 Chuo, Kurashiki, Okayama 710-0046 Japan

**Keywords:** Alpine plants, Dispersal, Genetic divergence, East Asia, Phylogeography

## Abstract

Mountain regions are important places for biodiversity, where organisms could persist throughout prolonged periods and accumulate genetic divergence as well as promote speciation. Roles of mountains for biodiversity have been exclusively discussed in regions that have specifically diverse species or covered with ice-sheets during the Pleistocene glacial periods, whereas the importance of mountainous regions in East Asia has been less disputed. High mountains in the Japanese Archipelago, located at the eastern edge of the Eurasia continent, have one of southernmost populations of alpine and arctic-alpine plants that are also distributed in the northern Pacific and/or the circumarctic regions. Phylogeographic studies on the Japanese alpine plants have excluded their possible ephemeral occurrence during the current warm period, and rather, suggest persistence of alpine plants throughout several cycles of climate changes in the Pleistocene on high mountains in central Honshu, the main island of the Japanese Archipelago. In this review, I look through decade long phylogeographic studies and show complicated patterns of range dynamics of Japanese alpine plants. In addition, I note recent findings of genetic relationships of Japanese populations of alpine and/or arctic-alpine plants with those in northern regions and their possible ecological divergence in the Japanese Archipelago. Taken together, I provide several issues for understanding historical processes that established distribution of alpine plants following climate changes as well as their diversification and propose importance of Japanese populations of alpine plants on biodiversity in alpine communities across broader range, especially in the northern Pacific region.

## Introduction

Mountain regions harbour a high terrestrial biodiversity (Rahbek et al. 2019b), where topographic complexity and elevational climatic gradients not only enhance divergence as well as speciation but also maintain diverse species (Badgley et al. 2017; Rahbek et al. 2019a; Rangel et al. 2018). Although higher diversity is usually observed in lower-latitude mountains than higher-latitude one (Rahbek et al. 2019b), mountains in temperate and arctic regions play important roles for terrestrial biodiversity as well. In particular, mountains in East Asia were not covered with continental ice-sheets in the Pleistocene, enabling mountainous species including alpine and arctic-alpine plants to persist throughout repeated cycles of warm and cold periods as refugia and accumulate unique genetic divergence (Hewitt 1996, 2000, 2004). Furthermore, the northern part of East Asia is adjacent to the southwest of Beringia, an ice-free area around Bering Straits having been noted as an important refugium of cold-adapted species during the Pleistocene glacial periods (Abbott 2000; Abbott and Brochmann 2003; Hultén 1937). As a consequence, cold-adapted species could have spread their range southward from Beringia into north-eastern Asia and vice versa following climate changes in the Pleistocene. Although numerous studies have emphasized importance of Beringia as refugia as well as sources of postglacial range expansion for various cold-adapted (Abbott 2000; Abbott and Brochmann 2003; Shafer et al. 2010), range dynamics of cold-adapted species between Beringia and north-eastern Asia has been barely disputed.

**Fig. 1:**
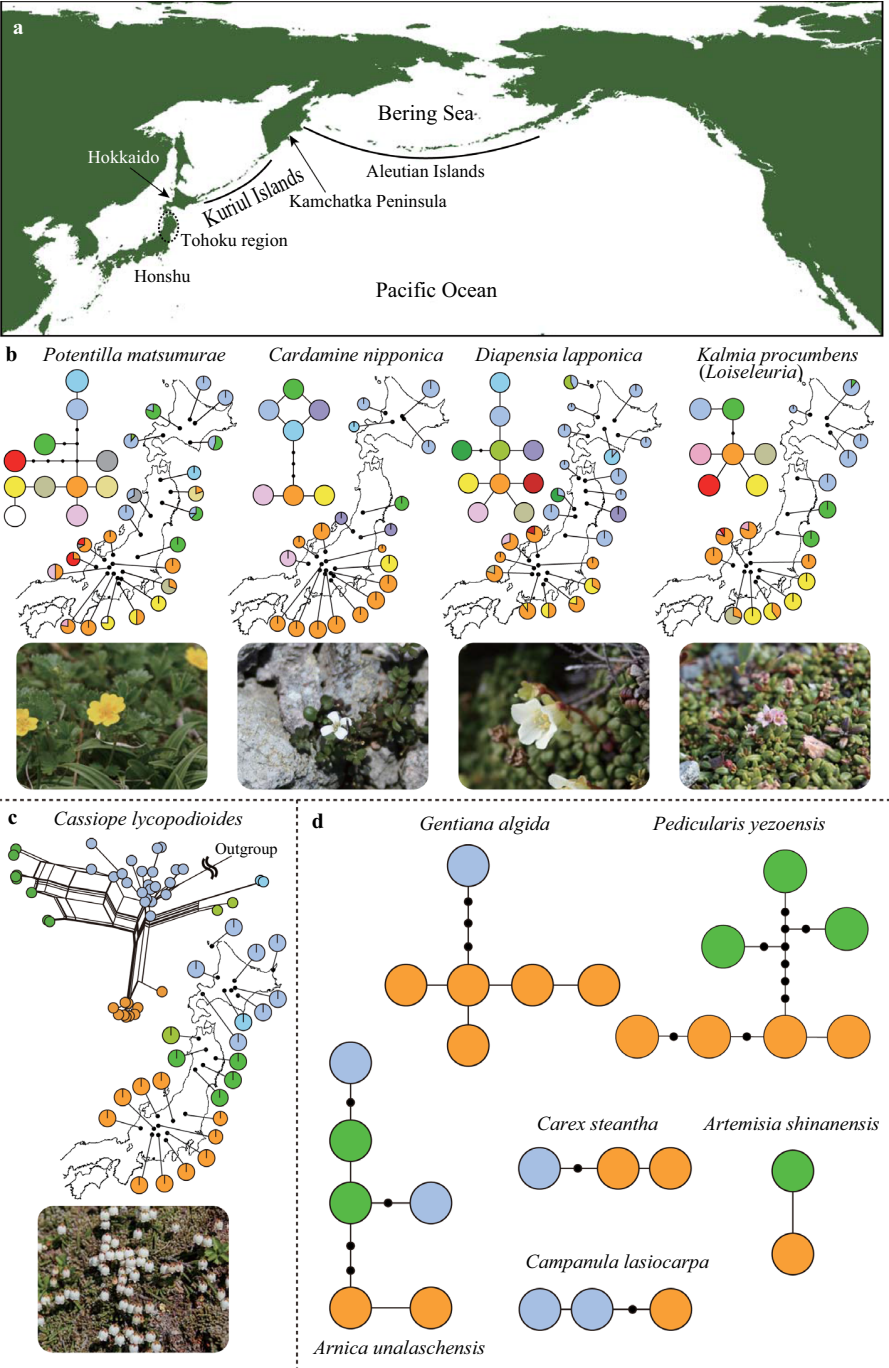
Genetic structure of alpine plants in Japan with clear genetic differentiation between the central part of the main island (Honshu) and northern Japan. **a** The location of Japan. Alpine plants mainly occur on high mountains in central Honshu and Hokkaido. **b** Distributions of plastid DNA (pDNA) haplotypes and their phylogenetic network in four species (*Potentilla matsumurae*, *Cardamine nipponica*, *Diapensia lapponica*, and *Kalmia procumbens*). Pie charts reflected haplotype frequencies in each population, where colours reflect those in network. **c** Distribution of major clusters based on multiple nuclear loci and phylogenetic networks among individuals in *Cassiope lycopodioides*. Colour circles in the network corresponding to the cluster, to which each individual was assigned. **d** Haplotype networks in six species (*Gentiana algida*, *Pedicularis yezoensis*, *Arnica unalaschensis*, *Carex steantha*, *Campanula lasiocarpa*, and *Artemisia shinanensis*). Orange, green and blue circles indicate haplotypes that occurred in central Honshu, Tohoku region, and Hokkaido, respectively. The genetic data originated from citations in Table 1

The Japanese Archipelago is located at the eastern edge of the Eurasia continent and stretches approximately 3000 km from north to south. The northern part of the archipelago is adjacent to Kuril Islands that stretch northeast to Kamchatka Peninsula, a part of Beringia. The Japanese Archipelago has mountains as high as *ca*. 3000 m, especially in the central part of the main island (Honshu; Fig. 1a). Although these mountains are too warm to have glaciers at present, strong wind and/or plenty of snow limits forest expansion on higher part of mountains. Instead, high mountains in the Japanese Archipelago are occupied by alpine communities, which harbour *ca*. 440 species of alpine plants (Shimizu 1982, 1983). Approximately 43% of them are the species or closely related species that are distributed in northern regions such as the northern Pacific region and the Arctic, while the remaining are closely related to species in the Eurasian continent or those at lower elevations in Japan. Arctic-alpine plants including *Empetrum nigrum* L., *Diapensia lapponica* L., and *Kalmia procumbens* (L.) Gift et Kron et P. F. Stevens ex Galasso, Banfi et F. Conti mostly dominate in the plant communities on the high mountain ridges, whereas snow-bed species occurring in the northern Pacific region including *Phyllodoce aleutica* (Spreng.) A. Heller, *Primula cuneifolia* Ledeb., and *Sieversia pentapetala* (L.) Greene form large communities at moist part of mountains covered with snow until late summer. Accordingly, alpine plants in the Japanese Archipelago have long been recognized to have originated by cold-adapted species in northern regions that spread southward during the Pleistocene cold periods and have survived climatic warming on high mountains (Koidzumi 1919).

Given such relict occurrence of Japanese alpine plants, one may suppose that alpine plants form ephemeral populations in Japan during the current warm period. However, phylogenetic studies revealed the genetic divergence of alpine plants in central Honshu and suggest that alpine plants have persisted there for prolonged period in the Pleistocene (Fujii and Senni 2006). Such refugial persistence of alpine plants was further emphasized by comparatively high genetic diversity of alpine plants across numerous plants including montane and lowland species (Ohsawa and Ide 2011). As a consequence, prolonged persistence in central Honshu is a typical history of alpine plants in Japan. In contrast, recent studies have not only shown more complicated patterns of genetic structure within Japan but also inferred range shifts of alpine plants beyond the Japanese Archipelago, especially encompassing around the northern Pacific region.

In this review, by looking through decades long phylogeographic studies on Japanese alpine plants, I show four geographic patterns of alpine plants observed in Japan and note unaddressed issues underlying the genetic structure. In addition to the exclusive focus on the Japanese Archipelago, I discuss historical scenarios of range shifts that alpine plants experienced across north-eastern Asia as well as the northern Pacific region. Furthermore, I note possible ecological divergence of alpine plants in the Japanese Archipelago and its importance on biodiversity across a broad range.

## Genetic divergence between northern and central Japan

**Fig. 2:**
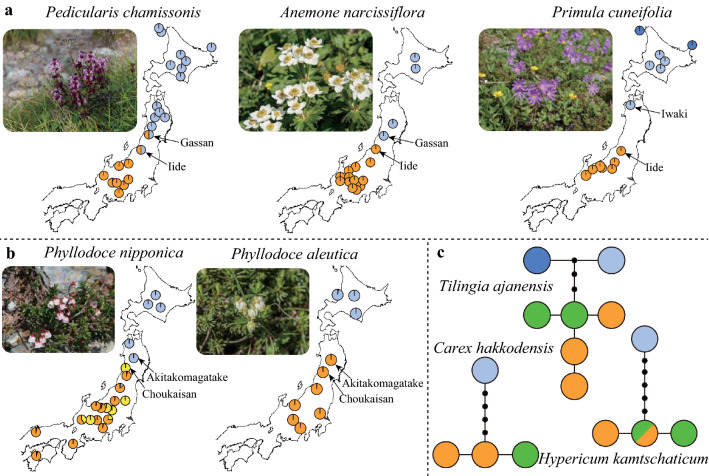
Genetic structure of alpine plants in Japan that have lacks unique genetic divergence in central Honshu. **a** Distribution of major haplotype groups in three species (*Pedicularis chamissonis*, *Anemone narcissiflora*, *Primula cuneifolia*). Each colour reflects different clades of haplotypes. **b** Distribution of genetic clusters based on multiple nuclear loci in *Phyllodoce nipponica* and *Phyllodoce aleutica*. **c** Haplotype networks in three species (*Tilingia ajanensis*, *Carex hakkodensis*, and *Hypericum kamtschaticum*). Orange, green and blue circles indicate haplotypes that occurred in central Honshu, Tohoku region, and Hokkaido, respectively. The genetic data originated from citations in Table 1

Genetic structure of Japanese alpine plants was firstly investigated by intraspecific phylogenetic analyzes of *Pedicularis chamissonis* Steven (Orobanchaceae), which has small seeds with moderate dispersal ability in capsule, using three inter genetic spacers of plastid DNA (pDNA; Fujii et al. 1997). This study found that individuals in central Honshu had haplotypes exclusively belonging to a clade (southern clade), while those in northern Japan had haplotypes in the other clade (northern clade). The similar genetic structure was further reported in *Primula cuneifolia* Ledeb (Primulaceae; Fujii et al. 1999), which has also small seeds with moderate dispersal ability in capsule. By investigating intraspecific phylogenies using three intergenic spacers of pDNA, they revealed that a highly supported clade was formed by haplotypes that exclusively occurred in central Honshu as well as Mt. Iide (Fig. 2a) in the southern part of the northern Honshu (Tohoku region). Although *P. cuneifolia* did not have a monophyletic group of haplotypes in northern regions including the Kuril islands, the Kamchatka Peninsula, and the Aleutian Islands as *P. chamissonis* did, *P. cuneifolia* in central Honshu was genetically distinguished from those in northern Japan.

According to the genetic structure, earlier studies proposed a scenario of range dynamics in Japanese alpine plants (Fujii et al. 1997, 1999; Senni et al. 2005). Unique haplotypes in central Honshu reflected the earlier migrants that colonized during a Pleistocene glacial period and have persisted there throughout the Pleistocene climate changes as refugia during interglacial periods. On the other hand, haplotypes in northern Japan reflected the latest southward migration, which spread from northern regions but did not reach central Honshu. Thus, the range expansion during at least two different Pleistocene glacial periods established current distributions of Japanese alpine plants and shaped their genetic structure.

Further phylogenetic studies revealed the similar north–south divergence in three species (*Anemone narcissiflora* L., *Cardamine nipponica* Franch. et Sav., and *Kalmia* [syn. *Loiseleuria*] *procumbens*) (Fujii and Senni 2006). These findings emphasized the north–south divergence as the common pattern of genetic structure in Japanese alpine plants. Furthermore, phylogeographic studies analyzed distribution patterns of pDNA haplotypes of a number of samples and demonstrated genetic differentiation between populations in central Honshu and northern Japan in *Potentilla matsumurae* Th.Wolf (Ikeda et al. 2006)*, Diapensia lapponica* L. (Ikeda et al. 2008c), *K. procumbens* (Ikeda et al. 2009c). Moreover, the north–south differentiation was further demonstrated by nuclear markers using AFLP in *P. matsumurae* (Ikeda et al. 2008b) and sequences of multiple nuclear loci in *C. nipponica* (Ikeda et al. 2008a), *Phyllodoce nipponica* Makino (Ikeda and Setoguchi 2013), *Cassiope lycopodioides* (Pall.) D.Don (Ikeda et al. 2014), *K. procumbens* (Ikeda et al. 2017) and *Phyllodoce aleutica* (Spreng.) A.Heller (Ikeda et al. 2018). In addition, preliminary investigations revealed that individuals in central Honshu and northern Japan had different haplotypes in other nine species [*Arnica unalaschcensis* Less., *Artemisia sinanensis* Y.Yabe, *Carex stenantha* Franch. et Sav., *Campanula lasiocarpa* Cham.*, Gentiana algida* Pall., *Pedicularis yezoensis* Maxim.*, Carex hakkodensis* Franch.*, Hypericum kamtschaticum* Ledeb.*,* and *Tilingia ajanensis* Regel] (Senni et al. 2005). Hence, 19 species from 13 families exhibited the genetic divergence between northern and central Japan (Type Ia, Ib, and Ic in Table 1 and Figs. 1 and 2).

**Table 1:** Phylogeographic studies on alpine plants in Japan

Species	Family	References	Markers	No. population	No. samples
Type Ia: North–south divergence (Divergence between C Honshu and N Japan)					
*Arnica unalaschcensis*	Asteraceae	**Senni et al.** (2005)	pDNA (ca. 2020 bp)	9	9
*Artemisia sinanensis*	Asteraceae	**Senni et al.** (2005)	pDNA (ca. 1810 bp)	8	8
*Campanula lasiocarpa*	Campanulaceae	**Senni et al.** (2005)	pDNA (ca. 1930 bp)	8	8
*Cardamine nipponica*	Brassicaceae	Senni et al. (2005)	pDNA (ca. 1940 bp)	10	10
		Fujii and Senni (2006)	pDNA (5 regions)	14	14
		**Ikeda et al.** (2008a)	pDNA (750 bp) + nDNA (3 loci)	19	279
*Carex stenantha*	Cyperaceae	**Senni et al.** (2005)	pDNA (ca. 1830 bp)	10	10
*Cassiope lycopodioides*	Ericaceae	Senni et al. (2005)	pDNA (ca. 2630 bp)	8	8
		**Ikeda et al.** (2014)	nDNA (12 loci)	24	83
*Diapensia lapponica*	Diapensiaceae	Senni et al. (2005)	pDNA (ca. 2260 bp)	11	11
		**Ikeda et al.** (2008c)	pDNA (ca. 1130 bp)	22	159
*Gentiana algida*	Gentianaceae	**Senni et al.** (2005)	pDNA (ca. 2200 bp)	8	8
*Kalmia (Loiseleuria) procumbens*	Ericaceae	Senni et al. (2005)	pDNA (ca. 1810 bp)	9	9
		Fujii and Senni (2006)	pDNA (5 regions)	15	15
		**Ikeda et al.** (2009c)	pDNA (ca. 850 bp)	17	152
		Ikeda et al. (2017)	nDNA (12 loci)	40	73
*Pedicularis yezoensis*	Orobanchaceae	**Senni et al.** (2005)	pDNA (ca. 2460 bp)	10	10
*Potentilla matsumurae*	Rosaceae	Senni et al. (2005)	pDNA (ca. 1950 bp)	11	11
		**Ikeda et al.** (2006)	pDNA (ca. 1400 bp)	22	203
		Ikeda et al. (2008b)	pDNA (ca. 1400 bp)	22	161
Type Ib: North–south divergence (Divergence around Iide)					
*Pedicularis chamissonis*	Orobanchaceae	**Fujii et al.** (1997)	pDNA (ca. 1760 bp)	24	55
*Anemone narcissiflora*	Ranunculaceae	Senni et al. (2005)	pDNA (ca. 1880 bp)	13	13
		**Fujii and Senni** (2006)	pDNA (5 regions)	17	17
*Primula cuneifolia*	Primulaceae	**Fujii et al.** (1999)	pDNA (ca. 1870 bp)	20	97
Type Ic: North–south divergence (Divergence in more northern part in Tohoku or between Tohoku and Hokkaido)					
*Carex hakkodensis*	Cyperaceae	**Senni et al.** (2005)	pDNA (ca. 1840 bp)	8	8
*Hypericum kamtschaticum*	Hypericaceae	**Senni et al.** (2005)	pDNA (ca. 1640 bp)	12	12
*Tilingia ajanensis*	Apiaceae	**Senni et al.** (2005)	pDNA (ca. 1840 bp)	9	9
*Phyllodoce aleutica*	Ericaceae	**Ikeda et al.** (2018)	nDNA (13 loci)	16	84
*Phyllodoce nipponica*	Ericaceae	Ikeda and Setoguchi (2007)	pDNA (ca. 1450 bp)	19	155
		**Ikeda and Setoguchi** (2013)	pDNA (ca. 580 bp) + nDNA (5 loci)	22	66
Type II: homogenous patterns					
*Carex scita*	Cyperaceae	Senni et al. (2005)	pDNA (ca. 1840 bp)	6	6
*Geranium yesoense*	Geraniaceae	Senni et al. (2005)	pDNA (ca. 930 bp)	5	5
*Geum calthifolium*	Rosaceae	Senni et al. (2005)	pDNA (ca. 2800 bp)	11	11
*Luzula arcuata*	Juncaceae	Senni et al. (2005)	pDNA (ca. 1990 bp)	8	8
*Minuartia (Arenaria) arctica*	Caryophyllaceae	Senni et al. (2005)	pDNA (ca. 970 bp)	8	8
*Rumex montanus (Rumex alpestris)*	Polygonaceae	Senni et al. (2005)	pDNA (ca. 1560 bp)	5	5
*Sieversia pentapetala*	Rosaceae	Senni et al. (2005)	pDNA (ca. 1980 bp)	12	12
*Trollius riederianus*	Ranunculaceae	Senni et al. (2005)	pDNA (ca. 1940 bp)	10	10
*Arcterica nana*	Ericaceae	Ikeda and Setoguchi (2006)	pDNA (ca. 1070 bp)	13	193
		Ikeda and Setoguchi (2009)	AFLP (165 loci)	21	176
*Shizocodon soldanelloides*	Diapensiaceae	Higashi et al. (2013)	pDNA (ca. 1670 bp) + AFLP (189 loci)	48	384
*Vaccinium uliginosum*	Ericaceae	Hirao et al. (2011)	pDNA (ca. 1100 bp)	6	34
*Vaccinium vitis-idaea*	Ericaceae	Ikeda et al. (2015)	pDNA (ca. 340 bp) + nDNA (3 loci)	65	130
*Dryas octopetala*	Rosaceae	Hirao et al. (2017)	pDNA(ca. 1234 bp) + nDNA (1 locus) + nSSR (10 loci)	18	413

Genetic markers (Markers) and analyzed number of samples (No. population and No. samples) of each publication are shown. Bold reference indicates citations with original data for genetic structures in Figs. 1 and 2

## Issues on genetic boarders between northern and southern groups

The prevalence of unique genetic variation in central Honshu implies that the earlier scenario of range dynamics would be applicable for various alpine plants. In particular, the clear differentiation between populations in central Honshu and northern Japan (including Iide and more northern region) strongly supported the scenario of colonization history occurring at least twice during different glacial periods. Such scenario was likely shared among 11 species including five preliminary investigated species (Type Ia in Table 1, Fig. 1). However, the genetic border around Iide is unclear in some species. For example, *Pedicularis chamissonis* exhibited the same pattern with Type Ia except for genetic admixture at the boarder region, where both northern and southern haplotypes occurred in Iide as well as Gassan (Fig. 2a)(Fujii et al. 1997). *Anemone narcissiflora* had southern haplotype in Iide (Fig. 2a)(Fujii and Senni 2006). Occurrence of southern haplotypes in Iide was also observed in *P. cuneifolia* (Fujii et al. 1999), while distribution of this species in Tohoku region is solely limited in Iide and Iwaki (Fig. 2a).

In addition, the other pattern of genetic structure was observed in the remaining species (Type Ic in Table 1). *Phyllodoce nipponica* was differentiated between Akitakomagatake and Choukaisan (Ikeda and Setoguchi 2013), whereas *P. aleutica* was between Honshu and Hokkaido (Ikeda et al. 2018) (Fig. 2b). The similar pattern of genetic divergence in Tohoku region was also reported by the preliminary study of pDNA in three species (*C. hakkodensis*, *H. kamtschaticum*, *T. ajanensis*) (Fig. 2c, Table 1), whereas it remains unclear whether there are shared patterns of location of genetic gaps among these species. Hence, the genetic divergence does not necessarily reflect a single physical barrier that commonly prevented dispersal of various species.

The scenario with prolonged refugia in central Honshu implies that alpine plants could spread their range in Japan during the last glacial period both by southward colonization from northern regions and northward expansion from refugia in central Honshu. In this case, strong barriers for dispersal between Iide and central Honshu may result in the unambiguous genetic differentiation, whereas the lack of such barriers may either complicate the location of genetic borders or cause genetic admixture around Iide and Gassan due to secondary contact of northward and southward migrants. Given that mountains in Tohoku region as well as between Iide and central Honshu are mostly lower in elevation (< 2500 m) compared to central Honshu (ca. 3000 m), species exclusively occurring in higher part of mountains such as *C. nipponica* and *K. procumbens* (Fig. 1b) have limited distribution in Tohoku region. As a consequence, dispersal of these species were highly restricted in Tohoku regions, resulting in the unambiguous genetic differentiation. In contrast, species that occur in rather low part of mountains may have easily dispersed between central Honshu and Iide, exhibiting various patterns of genetic borders as well as genetic admixture. For example, *P. chamissonis* and *A*. *narcissiflora* occur in meadows on mountain slopes, and therefore have wider range including at lower elevation. *Phyllodoce nipponica* has wider range encompassing the western Japan, where elevation of mountains is comparatively low (< 2000 m) and only a few alpine plants are distributed (Fig. 2b). Thus, further investigations considering habitat preferences are crucial to address the discrepancy in genetic structure at Tohoku region.

## Temporal frameworks underlying the genetic structure

Comparing geographic patterns of genetic variation enables us to infer a shared history of prolonged refugial isolation and/or postglacial range expansion. In particular, comparative phylogeography would be an efficient approach to identify locations of refugia during the last glacial period and infer postglacial dispersal processes in various temperate and alpine species in Europe and North America (Alsos et al. 2007; Eidesen et al. 2013; Schönswetter et al. 2005; Shafer et al. 2010; Soltis et al. 2006). Although most of these studies focused on species whose range was mostly covered with ice-sheets in the Pleistocene glacial periods, alpine plants in the Japanese Archipelago were not completely erased by the last glacial period. Rather, they could spread in Japan during glacial (cold) periods and persisted in some current populations throughout the Pleistocene climate changes as refugia during interglacial (warm) periods. Such a prolonged history in Japan has made it possible that different historical events including migration or range separation occurring during different periods have converged into a similar genetic structure.

To address this issue, estimating temporal framework of genetic divergence would be crucial. The early studies inferred that the divergence of Japanese clades in *P. chamissonis* and *P. cuneifolia* predated the middle of Pleistocene and dated back to the middle of Pliocene (3,800–400 ka; Table 2). In contrast to these estimations, recent model-base analyzes estimated more recent divergence in five species (Table 2). Among these studies, the divergence between northern and southern groups likely occurred during the last interglacial period in *C. nipponica* and *C. lycopodioides* (ca. 106 ka; Table 2) (Ikeda et al. 2009a, 2014). In addition, the last interglacial divergence is a plausible history for the genetic differentiation in *P. aleutica* as well (Ikeda et al. 2018). Furthermore, the divergence following refugial isolation could be applicable for *P. nipponica* as well, whereas its divergence occurred prior to the last interglacial period (249.0–180.2 ka; Table 2)(Ikeda and Setoguchi 2013). These inferences are consistent with the range separation as well as refugial isolation between northern and southern populations in Japan during the interglacial period(s), one in central Japan and the other in northern region.

**Table 2:** Summary of divergence time between central Honshu and northern Japan in seven alpine plants

Species	*T* (ka)	Markers	Substitution rates (*10^–9^)	References
(a) Genetic distance				
*Pedicularis chamissonis*	3800–600	pDNA (ca. 1760 bp)	1.30–8.24	Fujii and Senni (2006)
*Primula cuneifolia*	3000–470	pDNA (ca. 1870 bp)	1.30–8.24	Fujii and Senni (2006)
(b) IM model				
*Kalmia procumbens*	64.8–43.9 (113.4–21.7)	nDNA (12 loci)	5.30–7.80	Ikeda et al. (2017)
*Phyllodoce aleutica*	82.0–56.4 (140.4–30.0)	nDNA (13 loci)	5.30–7.80	Ikeda et al. (2018)
*Cardamine nipponica*	106.0 (3,565–38.0)	nDNA (10 loci)	8.67	Ikeda et al. (2009a)
*Cassiope lycopodioides*	106.9 (257.6–51.4)	nDNA (9 loci)	6.00	Ikeda et al. (2014)
*Phyllodoce nipponica*	249.0–180.2 (474.7–58.5)	nDNA (12 loci)	6.00	Ikeda and Setoguchi (2013)

The divergence time (*T*) are shown by the scaled divergence time based on genetic distance (a) or the maximum likely estimation with 95% highest posterior densities in parenthesis estimated by an isolation with migration (IM) model (b). Genetic markers (Markers) and assumed substitution rates (Substitution rates) are shown together with each reference

On the contrary, *K. procumbens* in central Honshu likely diverged during the last glacial period (82–43 ka; Table 2) (Ikeda et al. 2017), which may represent its southward colonization into central Honshu during the last glacial period. This implies that the north–south divergence of Japanese alpine plants did not necessarily reflect a shared isolation history among various species but was possibly shaped by either interglacial refugial isolations or glacial range expansion.

It should be noted that these estimations depend on an assumption of substitution rates, where all studies did not apply a specific rate for each species. In addition, difference in generation times, especially between both shrub and herb species, might influence the divergence time. Furthermore, confidence intervals of each estimation usually encompassed several glacial and interglacial periods. Addressing these limitations would be crucial for robust inference of historical events underlying genetic structure. Nevertheless, given that the latest events of range dynamics have more influenced genetic structure than earlier events, the range separation during the last interglacial period or southward colonization during the last glacial period are reasonable scenarios.

## Homogenous genetic pattern throughout Japan

Although phylogeographic studies have exclusively focused on the genetic divergence in between northern and southern Japan, all Japanese alpine plants did not necessarily exhibit genetic structure across populations in Japan (Type II in Table 1). A phylogeographic study on *Arcterica nana* (Maxim.) Makino provides the first sufficient evidence for the lack of genetic structure in pDNA haplotypes (Ikeda and Setoguchi 2006), which detected two widespread haplotypes across the Japanese populations. This homogenous pattern of genetic variation was further supported by AFLP study (Ikeda and Setoguchi 2009), suggesting that its Japanese populations have been established by a single range expansion during the last glacial period.

The lack of genetic divergence has been also reported in *Schizocodon soldanelloides* Siebold et Zucc. (Higashi et al. 2013), an endemic perennial herb species to Japan occurring both on high mountains and in understory of deciduous forest. Although its haplotype frequencies differed between western and northern Japan, this species lacks any structured pattern of genetic divergence in AFLP. Interestingly, ENM predicted that *S. soldanelloides* has a much smaller potential distribution mostly at central Japan during the LGM than at the present time. Thus, postglacial northward and southward range expansion plausibly established the current distribution as well as the geographically homogenous pattern of genetic variation in *S*. *soldanelloides*.

In addition to these extensive studies, the lack of genetic divergence in Japan was reported in other species (Table 1) including *Vaccinium vitis-idaea* L. (Ikeda et al. 2015), *Vaccinium uliginosum* L. (Hirao et al. 2011), and *Dryas octopetala* L. (Hirao et al. 2017). Furthermore, preliminary investigations revealed that other seven species had a widespread pDNA haplotype in both central Honshu and northern Japan (Senni et al. 2005), potentially reflecting the lack of genetic structure. These species may have spread their range across Japan by a single dispersal event during the last glacial or postglacial period. For example, the southward colonization during the last glacial period would be a more probable event for arctic-alpine species including *V. vitis-idaea*, *V. uliginosum* and *D. octopetala*. On the other hand, as inferred in *S. soldanelloides*, both northward and southward range expansion was an alternative scenario underlying the homogenous genetic structure. Given that *S. soldanelloides* also grows in forest understory in lower elevation, species occurring both in higher and lower parts of mountains in Japan might have recently expand their range throughout the Japanese Archipelago. Since previous phylogeographic studies exclusively focused on species with the north–south divergence (Fujii and Senni 2006; Ikeda et al. 2008c, 2009c), it remains to be explored whether the north–south divergence is common or rare across species in Japanese alpine communities. Although phylogeographic studies based on a small number of loci has been somewhat insufficient to distinguish the lack of genetic structure due to low genetic variation, this issue will be challenged by genome-wide studies using next generation sequences (NGS).

## Range dynamics beyond the Japanese Archipelago

Recent studies have revealed genetic variation of alpine plants in Japan together with their populations from more widespread ranges encompassing the northern Pacific region. These studies have emphasized genetic uniqueness in central Honshu (*Cassiope lycopodioides*, Ikeda et al. 2014; *Kameria procumbens*, Ikeda et al. 2017; *Phyllodoce aleutica*, Ikeda et al. 2018; *Primula cuneifolia*, Ikeda et al. 2020), supporting their in situ persistence. Furthermore, they revealed genetic similarity between northern Japan and more northern regions, especially the northern Pacific region (Hata et al. 2017; Ikeda et al. 2014, 2017, 2018, 2020). These findings suggest three possible scenarios of the latest range dynamics of alpine plants: alpine plants (i) were continuously distributed across northern Japan and the northern Pacific region during the last glacial period, from which their range contracted following postglacial climate warming, (ii) spread northward from northern Japan or neighbouring regions into the northern Pacific region, or (iii) spread southward from Beringia to northern Japan.

Phylogeographic studies on *Phyllodoce aleutica* (Ikeda et al. 2018) and *Primula cuneifolia* (Ikeda et al. 2020) supported the second scenario. *Phyllodoce aleutica* in Alaska has little genetic diversity across its extensive range, where the genetic variation reflects a demographic model postulating the postglacial range expansion (Ikeda et al. 2018). This finding indicates that *P. aleutica* recently colonized Alaska from northern East Asia via the Aleutian Islands. *Primula cuneifolia* in central Honshu belonged to a clade that diverged earlier than clades in northern Japan, Kamchatka Peninsula, and Alaska. In addition, these intraspecific clades diverged during and after the last glacial period, suggesting that *P. cuneifolia* in the northern Pacific region originated by the northward migration from central Honshu (Ikeda et al. 2020).

The northern Pacific region was a part of the Beringia refugium, where a large ice-free area enabled cold-adapted species to survive climate changes for prolonged periods in the Pleistocene. Range expansion from Beringia has been the most familiar scenario how cold adapted species spread their widespread ranges (Abbott and Brochmann 2003; Eidesen et al. 2013; Hultén 1937; Ikeda et al. 2017). On the contrary, the northward migration of alpine plants from Japan to the northern Pacific region provides novel insight: i.e., cold-adapted species distributed in the former Beringia have not necessarily persisted there rather some species colonized Beringia from north-eastern Asia during and after the last glacial period. Accordingly, alpine plant communities in the north-eastern Asia and the northern Pacific region would have been formed by species migrating from different regions, and thereby, have responded to climate changes in complicated manners. Further comparative studies incorporating numerous species could fully resolve the complicated range dynamics of alpine as well as arctic-alpine plants in northern Pacific region. For this issue, samplings from remote populations, especially at the Kamchatka Peninsula, the Kuril Islands, and the Aleutian Islands, are the important challenge as well.

Notably, the northward migration provided further issues in the range dynamics of alpine plants in Japan. Previous studies infer their range dynamics with the assumption that Japanese alpine plants migrated southward from northern regions around the Bering Sea during the Pleistocene glacial periods (Fujii and Senni 2006). As the genetic structure of *P. cuneifolia* (Ikeda et al. 2020) contradicts this assumption, careful assessments on direction and timing of migration are required for understanding range dynamics of alpine plants in Japan.

## Ecological divergence in the Japanese Archipelago

The refugial isolation in central Honshu and the subsequent southward colonization from northern regions could explain the north–south divergence of Japanese alpine plants. However, if this scenario is true, the latest southward migration, perhaps occurring during the LGM, may have either remained genetic footprints of the colonization or erased the pre-existing genetic structure in central Japan; thus, the present clear genetic structure is rather enigmatic. One possible explanation for the genetic structure is that southward migrations of alpine plants rarely occurred in Japan even during glacial periods, and thereby, the latter migrants may have little influence on the pre-existing genetic structure. Given a palaeobotanical inference that northern and central Honshu were mostly covered with boreal forest during the LGM (Tsukada 1983, 1985), the limited occurrence of alpine vegetation may have prevented alpine species from frequent dispersal across the Japanese Archipelago, making this hypothesis possible.

Alternatively, even if southward colonization occurred during the LGM, ecological factors may prevent genetic admixture between northern and southern groups, maintaining the genetic structure. Indeed, *Pedicularis chamissonis* had both northern and southern haplotypes in Gassan, whereas their distributions were highly structured within the mountain; i.e., a southern haplotype occurred in a higher part of the mountain slope with dry habitats than a northern haplotype that exclusively occurred in lower elevation with moist habitats. Moreover, corolla tubes of individuals with the northern type are curved, while those of a southern type are mostly straight (Fujii et al. 2001)*.* This morphological difference might be associated with pollinator-mediated premating reproductive isolation, where *P. chamissonis* is mainly visited by the bumblebee *Bombus beaticola* in central Honshu (Yumoto 1986) and in Hokkaido (Kawai and Kudo 2008). Thus, ecological divergence somewhat contributed to maintaining the genetic structure of *P. chamissonis*.

Furthermore, genetic footprints of divergent selection between the northern and southern populations suggest ecological roles for maintaining genetic structure. Ikeda et al. (2009b) investigated phylogeographic structure of *C. nipponica* using nuclear genes that are possibly involved in ecologically important roles in a model plant, *Arabidopsis thaliana*. Their study found that *PHYTOCHROME E* (*PHYE*), which encodes one of red and far-red light receptors, evolved with divergent selection, where amino acid replacements exclusively occurred on a domain for light perception (the PHY domain). In addition, a similar study on *A. nana* found that natural selection on amino acid changes resulted in clinal patterns of allele frequencies on *PHYE* between its northern and southern populations (Ikeda and Setoguchi 2010), whereas this species exhibited a homogenous pattern of genetic variation throughout Japan in other markers than *PHYE* (Ikeda and Setoguchi 2006, 2009). Interestingly, a recent study demonstrates that amino acid changes in phytochrome B (phyB), one of the other red and far-red light receptors, were responsible for divergence in light and temperature sensitivity between sister species growing in lower latitude (*C. nipponica*) and higher latitude (*Cardamine bellidifolia*) (Ikeda et al. 2021). Since phyB plays various roles in plant responses to environment signals throughout life cycle in *A*. *thaliana*, this finding implies that light and temperature sensitivity of phytochromes may be involved in ecological divergence between these sister species. In *A. thaliana*, phyE is involved in regulation of ecologically important traits such as flowering or seed germination at lower temperature (Halliday and Whitelam 2003; Heschel et al. 2007). Given that alpine and arctic-alpine plants possibly grow in warmer and more humid climate as well as shorter daylength and longer growing seasons in central Honshu than northern Japan, functional divergence of phyE might cause ecological divergence between northern and southern Japanese populations. Exploring functional divergence of phyE between northern and southern populations and examining whether the genetic footprints of natural selection on *PHYE* truly reflected divergence in ecologically important traits are required to address this issue. Since genome-wide investigations could examine natural selection through genomic scan (Luikart et al. 2003; Storz 2005), the phylogeographic studies incorporating genome-wide polymorphisms could not only infer demographic history underlying genetic structure but also provide ecological insight into the ecological process underlying divergence of Japanese alpine plants.

## Conclusive remarks

Decades long phylogeographic studies confirmed the importance of high mountains in central Honshu for alpine plants to persist throughout the climate changes in the late Pleistocene. On the contrary, these studies show that genetic uniqueness exclusively in central Honshu is not the single common pattern of genetic structure across Japanese alpine plants. Instead, there are four major patterns of genetic structure including the lack of genetic structure. Owing to the less glaciated history and subsequent possible prolonged persistence of alpine plants in the Japanese Archipelago, genetic structures of Japanese alpine plants were likely shaped by multiple historical events including migrations and/or range separation occurring during different periods. In addition, Japanese alpine plants contain species with various ecological characters as well as habitat preference, which may have influenced genetic structures as well. Thus, assessing demographic history as well as ecological characters is crucial to fully understand historical processes how alpine plants shaped the current distribution patterns in Japan.

Notably, the recent phylogeographic studies demonstrated that some alpine plants in Japan could have migrated northward, especially into the northern Pacific region, providing novel insight that alpine plants in Japan are not necessarily relict of cold adapted species distributed around Bering Straits. Further comparative studies could open new avenue for understanding the Pleistocene dynamics of alpine communities across broader ranges encompassing the northern Pacific region. Given potential ecological divergence of alpine plants within Japan, high mountains in Japan may have definitely played important roles for diversification of alpine plants not only in the archipelago but in northern East Asia as well as the northern Pacific region.
